# Risk of cognitive impairment with non-vitamin K antagonist oral anticoagulants in atrial fibrillation: Protocol for a systemic review of randomized controlled trials and real-world studies

**DOI:** 10.1097/MD.0000000000012072

**Published:** 2018-08-24

**Authors:** Long Shen, Chi Zhang, Zhi-Chun Gu, Hou-Wen Lin, Xiao-Yan Liu, Jun Pu

**Affiliations:** a Department of Cardiology; b Department of Pharmacy, Renji Hospital, School of Medicine, Shanghai Jiaotong University, Shanghai, China.

**Keywords:** atrial fibrillation, cognitive impairment, meta-analysis, non-vitamin K antagonist oral anticoagulants, real-world study

## Abstract

**Background:**

The association between use of non-vitamin K antagonist oral anticoagulants (NOACs) with cognitive impairment in atrial fibrillation (AF) remains unknown.

**Methods:**

An electronic search of Medline, Embase, Cochrane Library databases and ClinicalTrials.gov Website will be performed for randomized controlled trials (RCTs) that reported cognitive impairment events and observational nationwide database studies that reported adjusted hazard ratio (HR) in AF patients with NOACs. The primacy outcome will be a composite of any forms of cognitive impairment. HRs and their 95% confidence intervals (95% CI) will be calculated by using fixed- and random-effects models. Subgroup analyses will be undertaken based on individual NOACs, study types and follow-up duration.

**Results:**

This study will provide evidence of the association between use of NOACs and risk of cognitive impairment in patients with AF by pooling the results of RCTs and real-world studies.

**Conclusions:**

The results will bring about vigorous evidence in this topic and provide optimal anticoagulation strategy in AF patients at high risk of cognitive disorder.

**Ethics and dissemination:**

Ethical approval is not applicable for this study.

**PROSPERO registration number:**

CRD42018103849.

## 1 Introduction

Atrial fibrillation (AF) and cognitive impairment, such as dementia, are both frequent diseases, predominantly affecting the elderly. These 2 diseases are expected to be among the most prominent global epidemiological trends in the 21st century.^[1]^ AF and cognitive impairment share many common risk factors, including hypertension, diabetes mellitus, vascular disease and heart failure. Growing evidences reveal that AF is strongly associated with increased risk of cognitive disorder and dementia.^[2,3]^ The potential relationship between AF and cognitive impairment is not fully illuminated up to date. It is widely accepted that ischemic stroke is one of the key reasons leading to cognitive decline.^[1,4]^ Additionally, cerebral hypoperfusion, endothelial dysfunction or chronic inflammation are also involved in AF-related cognitive impairment.^[2]^

Oral anticoagulation is the cornerstone for the prevention of stroke in AF and is strongly recommended by guidelines.^[5]^ Whereas, the evidence of the association between anticoagulation and risk of cognitive impairment is limited. A recent study showed that warfarin-treated AF patients were at high risk of dementia due to under- or over-anticoagulation.^[6]^ Another observational study suggested that warfarin-therapy delays could increase the dementia risk in AF patients without a history of dementia.^[7]^ Therefore, it is plausible to make a hypothesis that warfarin might contribute to the preserved cognitive function due to the stroke prevention. Non-vitamin K antagonist oral anticoagulants (NOACs), targeting either thrombin or factor Xa, have been recommended as an optimal alternative due to their favorable property of thromboembolism prophylaxis and reduced bleeding risk in AF patients.^[8,9]^ Moreover, NOACs could offer low variability in anticoagulation effect, which leads to the hypothesis that NOACs might have a better protection against AF-related cognitive dysfunction than warfarin. Regretfully, limited information from randomized controlled trials (RCTs) have confirmed this hypothesis. When evidences from RCTs were insufficient to obtain conclusion, real-world studies could provide more valuable information. Hence, the present study will summarize current evidences of RCTs and high-quality real-world studies to carry out a meta-analysis regarding the association between NOACs and cognitive function in AF, as well as try to confirm this hypothesis.

## 2 Methods

### 2.1 Data sources and searches

This study will be conducted in accordance with PRISMA Statement and is registered in PROSPERO (registration number: CRD42018103849).^[10–12]^ Medline, Embase and Cochrane Library will be searched to identify potentially eligible studies with the following searching strategy: “dabigatran” or “Pradaxa” or “rivaroxaban” or “Xarelto” or “apixaban” or “Eliquis” or “edoxaban” or “Savaysa” or “betrixaban” or “Bevyxxa” or “Non-vitamin K antagonist oral anticoagulants” or “NOACs” or “direct oral anticoagulants” or “DOACs” or “novel oral anticoagulants” or “new oral anticoagulants” or “factor Xa inhibitors” or “factor II a inhibitors” in combination with “atrial fibrillation” or “AF”. Additionally, unpublished trials will be identified from the ClinicalTrials.gov Website. References of all pertinent articles will also be scrutinized to ensure that all relevant studies are identified.

### 2.2 Study selection and outcomes

The following inclusion criteria for study selection will be used: studies design must be RCTs or observational studies of NOACs reporting cognitive function outcomes; only high-quality nationwide or health insurance database studies reporting adjusted or matched results will be eligible; only study with the longest period will be included when different studies have applied the same data sources; studies that published only in abstract form or did not report adjusted results will be excluded. The primacy outcome is a composite of any cognitive dysfunction. The secondary outcomes are narrow definition of cognitive impairment (including dementia and Alzheimer's disease) and individual cognitive impairment reported in the study.

### 2.3 Data extraction, quality evaluation and bias assessment

Prespecified data variables will be extracted, including study characteristics, patient demographics, clinical characteristics, and cognitive function data. Detailed cognitive function data that is not reported in the original publications will be further extracted from the ClinicalTrials.gov website. Because cognitive impairment can be represented in various forms, the following outcomes will be used as cognitive impairment, which included amnesia, cognitive disorder, dementia, dementia Alzheimer's type, global amnesia, memory impairment, Parkinson's disease, Parkinsonism, vascular dementia, senile dementia, sensory disturbance, frontotemporal dementia, altered state of consciousness, amnestic disorder, and dementia with lewy bodies. The methodological quality of RCTs will be evaluated according to Cochrane Collaboration Risk of Bias Tool.^[13,14]^ The methodological quality of observational study will be evaluated with following domains: use of adjusted method to handle with selection bias; possibility of residual confounding; use of methods to deal with time-varying covariates; detailed reporting of baseline characteristics and outcome measures.^[15]^ Potential publication bias will be explored by using visual inspection of funnel plots.^[16,17]^

### 2.4 Data analysis

Hazard ratios (HRs) and their 95% confidence intervals (CIs) will be calculated using fixed- and random-effects models. Individual data of cognitive impairment will be merged, and corresponding HRs and CIs will be firstly calculated for RCTs. Then those observational studies reporting adjusted HRs and 95%CI will be pooled based on fixed- and random-effects models. Statistical heterogeneity will be assessed with *I*^2^ test, and *I*^2^ values of >50% represent considerable heterogeneity.^[18]^ Subgroup analyses will be conducted based on individual NOACs (dabigatran, rivaroxaban, apixaban, and edoxaban), study types (RCTs and database studies), and follow-up duration (> 1 year or <1 year). Meta-regression analysis will be performed to test demographic characteristics of the included studies. Sensitivity analyses will be carried out to assess the robustness of results with sequential elimination of individual studies. Further analyses will be conducted to identify the effect by excluding studies that involved catheter ablation, acetylsalicylic acid as control and low dosage arms of NOACs, or by adding the data of magnetic resonance imaging sub-study. All statistical analyses will be performed using STATA software (version13, Statacorp, College Station, TX), and *P* value of <.05 indicate a statistically significant difference.

## 3 Discussion

Effective anticoagulation reduces the burden of embolic stroke in AF patients and may bring preserved effects of cognitive function. NOACs, owing to favorable property of thromboembolism prophylaxis and reduced bleeding risk, have been recommended as an optimal alternative to warfarin. it is plausible to assume that NOACs might have a better protection against AF-related cognitive dysfunction than warfarin. For this hypothesis, we will perform a systematic review from RCTs and high-quality observational database studies to evaluate the association between use of NOACs and risk of cognitive impairment.

The relationship between AF and cognitive disorder might occur by a variety of pathological mechanisms. The leading potential mechanisms is the occurrence of stroke in AF patients, either overt or silent stroke. Stroke is recognized as the most feared complication in AF, and could be resulted from static blood produced by fibrillation of atrium, ultimately leading to thrombus formation and embolism to the brain.^[19,20]^ Of interest, silent cerebral infarction occurs more frequently than clinical stroke, and are more common in AF patients, which affect frontal circuit components that are essential for executive function.^[21]^ Vascular dementia may be another obvious contributor to cognitive impairment, including both multi-infarct dementia and small vessel disease dementia.^[22,23]^ In addition, low cardiac output in AF could lead to chronic cerebral hypoperfusion, which may in turn cause damage to the brain.^[19]^ Furthermore, AF was also identified to be a risk factor for Alzheimer's disease, the most common type of dementia.^[24,25]^

In terms of probable mechanisms described above, stroke prevention might lead to preserved cognitive function. Over the last decade, NOACs have been developed and revealed to be non-inferior to VKAs for the prevention of stroke, with a lower risk of intracranial micro-hemorrhage compared to warfarin.^[26,27]^ Regretfully, the question of whether NOACs could play a protective role on cognitive function in AF patients remains unanswered.

It is noteworthy that high-quality observational studies could bring more valuable evidence on additional risk of novel agents, especially when there are gaps in evidence from RCTs. A database study currently provided some optimism that AF patients with NOACs were associated with a lower risk of cerebral ischemic events and new-onset dementia than those with warfarin.^[26]^ For a strong argument on this issue, we will integrate RCTs and high-quality real-world studies to estimate the risk of cognitive impairment with NOACs.

Several possible limitations might be addressed in our study. Firstly, the inevitable heterogeneity between RCTs and observational studies need to be considered. Different confounding factors are adjusted in observational studies, which make it challenging to compare the results across the studies. Secondly, no included RCTs are especially designed to assess the cognitive function of NOACs, thus the absence of a clear and uniform definition of cognitive impairment and incomprehensive collection of cognitive data across trials might introduce certain bias. Thirdly, those excluded database studies not reporting the cognitive function data might reduce the power of statistics.

## 4 Conclusions

The results will bring about vigorous evidence in this issue and guide both clinical decision-making and future research.

## Author contributions

Jun Pu, Xiao-Yan Liu, and Hou-Wen Lin are the guarantors of the entire manuscript. Long Shen, Chi Zhang and Zhi-Chun Gu contributed to the study conception and design; data acquisition, analysis, and interpretation; drafting of the manuscript; critical revision of the manuscript for important intellectual content; and final approval of the version to be published.

**Conceptualization:** Long Shen, Chi Zhang, Zhi-Chun Gu, Hou-Wen Lin, Xiao-Yan Liu, Jun Pu.
